# Engineering hybrid microgels as particulate emulsifiers for reversible Pickering emulsions†

**DOI:** 10.1039/d1sc05398a

**Published:** 2021-10-29

**Authors:** Hang Jiang, Shengwei Zhang, Guanqing Sun, Yunxing Li, Xin Guan, Cheng Yang, To Ngai

**Affiliations:** The Key Laboratory of Synthetic and Biological Colloids, Ministry of Education & School of Chemical and Material Engineering, Jiangnan University Wuxi 214122 P. R. China yunxingli@jiangnan.edu.cn; Department of Chemistry, The Chinese University of Hong Kong Shatin, N. T. Hong Kong P. R. China tongai@cuhk.edu.hk

## Abstract

Thermo-responsive microgels are unique stabilizers for stimuli-sensitive Pickering emulsions that can be switched between the state of emulsification and demulsification by changing the temperature. However, directly temperature-triggering the phase inversion of microgel-stabilized emulsions remains a great challenge. Here, a hybrid poly(*N*-isopropylacrylamide)-based microgel has now been successfully fabricated with tunable wettability from hydrophilicity to hydrophobicity in a controlled manner. Engineered microgels are synthesized from an inverse emulsion stabilized with hydrophobic silica nanoparticles, and the swelling-induced feature can make the resultant microgel behave like either hydrophilic or hydrophobic colloids. Remarkably, the phase inversion of such microgel-stabilized Pickering emulsions can be *in situ* regulated by temperature change. Moreover, the engineered microgels were capable of stabilizing water-in-oil Pickering emulsions and encapsulation of enzymes for interfacial bio-catalysis, as well as rapid cargo release triggered by phase inversion.

## Abstract 2

Hybrid poly(*N*-isopropylacrylamide)-based microgels are templated from inverse Pickering emulsions, and the tunable wettability renders as-prepared emulsions with reversible feature.

## Graphical abstract

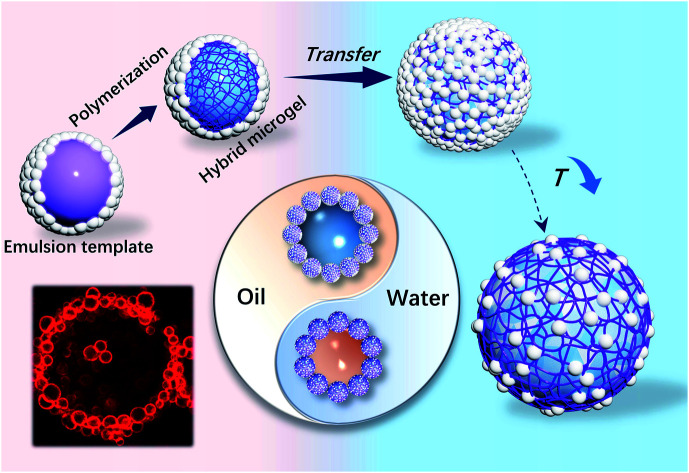

## Introduction

Microgels are soft and deformable colloid particles that can swell or shrink in a solvent with external stimuli.^1–4^ Touted as intelligent materials, microgels have received great attention^5,6^ and have been employed as rheology modifiers,^7,8^ delivery vehicles,^9^ emulsion stabilizers,^10,11^ micropattern templates,^12^ microreactors,^13^ and actuators.^14^ In particular, microgels are capable of spontaneously self-assembling at the oil/water interface and rapidly lowering the interfacial energy,^15–17^ and Ngai *et al.* pioneered the use of poly(*N*-isopropylacrylamide-*co*-methacrylic acid) (PNIPAM-*co*-MAA) microgels for stabilization of pH and temperature responsive oil-in-water (o/w) Pickering emulsions in 2004.^18^ Since then, numerous studies have been reported on microgel-stabilized emulsions^19^ with a variety of responses, including CO_2_/N_2_, sugar addition,^20^ magnetic field,^21^ and oxidation.^22^

Typically, microgels are highly hydrophilic and preferentially stabilize o/w Pickering emulsions.^23,24^ However, only forming o/w emulsions has application limitations, particularly in the preparation of aqueous-core microcapsules,^25^ interfacial bio-catalysis,^26–29^ gene delivery,^30^ and encapsulation of water-soluble actives.^31^ To address this issue, highly hydrophilic microgels were hydrophobized to some extent, allowing them to have an affinity for the oil phase and therefore stabilize water-in-oil (w/o) Pickering emulsions.^32^ In such cases, the phase inversion of microgel-stabilized emulsions from one type to another is conceivable, for example, inversion of o/w to w/o emulsions, which is becoming increasingly important in petroleum chemistry, drug delivery, and biphasic catalysis.^33–36^ Nevertheless, the previous reports of switchable Pickering emulsions stabilized by microgels were exclusively between the emlsification and demulsification, and directly triggering the phase inversion of microgel-stabilized emulsions remains a great challenge. For the purpose of reversion between o/w and w/o emulsions, microgels especially capable of being altered with sufficient hydrophobicity from the intrinsic hydrophilic state are required.

Watanabe *et al.* synthesized hydrophobized microgels for the first time to achieve stabilization of w/o Pickering emulsions with non-polar oils.^32,37^ By changing the composition of the hydrophobized microgel, both o/w and w/o Pickering emulsions can be generated. However, the reversion of the emulsion type from o/w to w/o was typically accomplished by increasing the grafted hydrophobic sites over the surface of microgels, *i.e.*, microgels hydrophobized at different levels were strategically selected. For *in situ* reversing the type of emulsion solely stabilized by microgels (without changing the composition of the microgel), the wettability of the microgel should be flexibly adjusted as hydrophobic or hydrophilic when the environment changes. Alternatively, coating the microgel with hydrophobic particles is an effective approach by taking advantage of expansibility of the microgel. When the microgel swells or collapses, the density of the hydrophobic particles on the surface decreases or increases, thus allowing the wettability of the microgel to be facilely adjusted.

Herein, we report a hybrid poly(*N*-isopropylacrylamide) (PNIPAM)-based microgel that was engineered for the fabrication of reversible Pickering emulsions. The surface of the PNIPAM microgel is coated with hydrophobic particles through an inverse Pickering emulsion template method, and the magnetic response is integrated by embedding Fe_3_O_4_ nanoparticles into the microgel. As a result, the hybrid microgel stabilized Pickering emulsion is able to be *in situ* switched between the o/w and w/o type. Furthermore, we show that the hybrid microgel in the hydrophobic state can make a breakthrough in the microgel-stabilized w/o Pickering emulsion for interfacial bio-catalysis. Moreover, an effective microencapsulation and burst release of water-soluble actives is realized *via* the phase inversion of the microgel-stabilized Pickering emulsion simply by hand shaking.

## Results and discussion

**Fig. 1:**
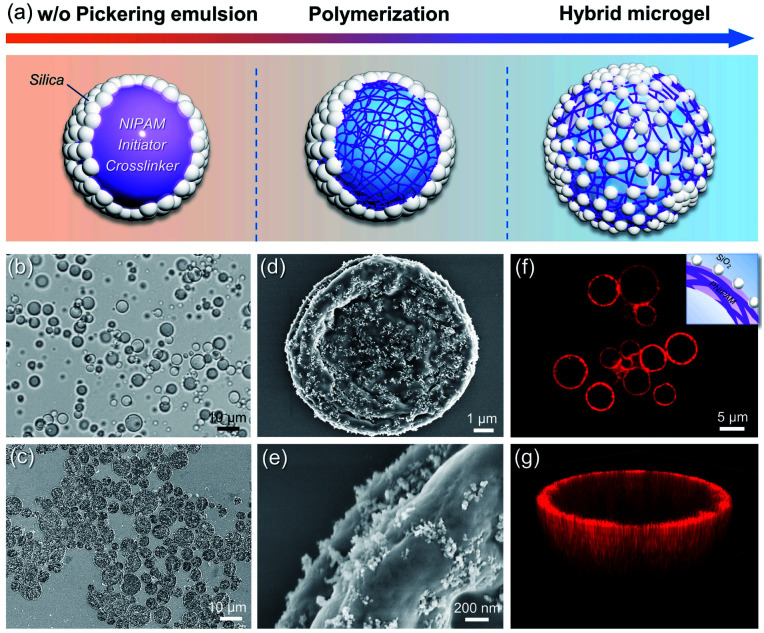
(a) Schematic illustration of the preparation of hybrid microgels. (b) Optical micrograph of the w/o Pickering emulsion template. (c–e) SEM images of the dried hybrid microgels. (f) CLSM image of the hybrid microgels and the inset shows the hollow structure of the microgel. (g) 3D reconstruction of the hybrid microgel dispersed in water *via* a z-stack of CLSM images. The red color indicates rhodamine B-labelled PNIPAM.

The schematic synthesis and morphology of the resulting hybrid microgels are depicted in Fig. 1. As illustrated in Fig. 1a, a w/o Pickering emulsion stabilized by hydrophobic silica nanoparticles (NPs) was prepared first, and the monomer, initiator, and crosslinker were all added in advance in the internal aqueous phase. Following polymerization, a hydrophobized hybrid PNIPAM microgel is expected to be formed from the w/o Pickering emulsion. Nearly irreversible adsorption of colloidal particles in Pickering emulsification and crosslinking polymer networks will make the silica nanoparticles coated on the microgel surface. These microgels were then collected and dispersed in water. Because of the change in the polymer-water affinity, the PNIPAM-based microgel swells in water below the volume phase transition temperature (VPTT = 32 °C) and shrinks above it.^38^ Optical micrographs of the w/o Pickering emulsion template (Fig. 1b and S1†) show that emulsion droplets had an average diameter of 4.2 μm, and in contrast, the average diameter of the resultant microgels dispersed in water increased to 7.5 μm, owing to water swelling (Fig. S2†). These hybrid microgels still retained sufficient softness and deformability, as a collapsed state could be observed after loss of water (Fig. 1c and d). And the surface of the microgel was relatively rough because of the distribution of hydrophobic silica NPs (Fig. 1e). Additionally, no free small NPs were observed, indicating that the subsequent Pickering emulsion was completely stabilized by hybrid microgels. It is worth noting that the confocal laser scanning microscopy (CLSM) observation revealed that the hybrid microgel had a hollow structure, with the shell composed of PNIPAM (Fig. 1f and g).

**Fig. 2:**
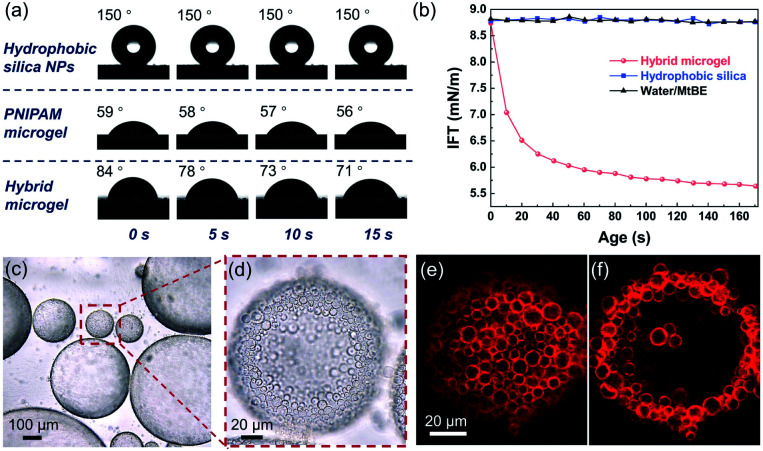
(a) Contact angles of a water drop on the films of the hydrophobic silica NPs, PNIPAM microgels, and hybrid microgels. (b) Dynamic interfacial tension between water and MtBE without (▲) or with the hybrid microgel (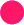) and hydrophobic silica NPs (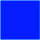). (c and d) Optical micrographs of the w/o Pickering emulsion stabilized by the hybrid microgels. (e and f) CLSM images of the hybrid microgels at the w/o interface observed from different focus points.

Contact angle measurements revealed that the hydrophobic silica NPs on the surface of the resulting hybrid microgel considerably enhanced its hydrophobicity. At room temperature, the contact angle of a water drop in air on a layer of hybrid microgel was approximately 90°, as illustrated in Fig. 2a. In comparison to the pure PNIPAM microgels synthesized by precipitation polymerization and the hydrophobic silica NPs, the dynamic contact angle of the hybrid microgel shows that it had intermediate wettability. Furthermore, when the hybrid microgel was applied, the dynamic interfacial tension between water and *tert*-butyl methyl ether (MtBE) was dramatically reduced (Fig. 2b), but the hydrophobic silica NPs alone had no noticeable effect, indicating that the hybrid microgel was interface active. Indeed, as seen in the optical microscopy and CLSM images of the w/o Pickering emulsion prepared at an oil/water volume ratio of 1 : 1 (Fig. 2c–f), the hybrid microgels with a hollow structure were closely stacked to form a dense layer at the w/o interface.

**Fig. 3:**
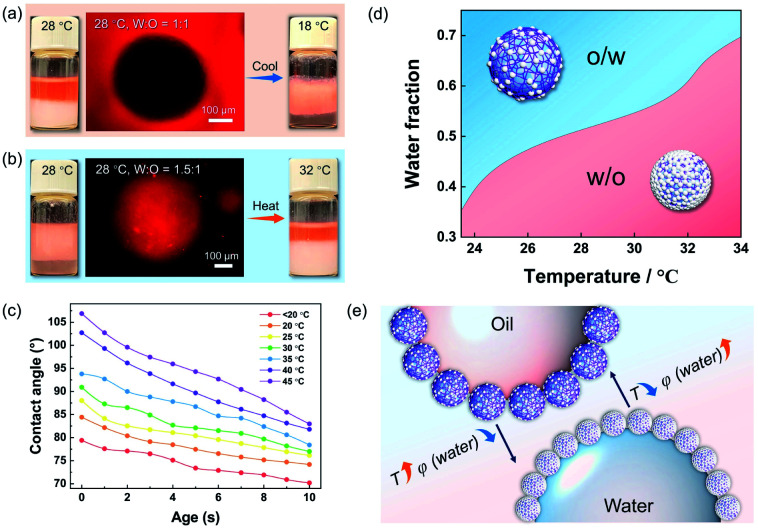
Photos and fluorescence microscopy images of Pickering emulsions formulated with hybrid microgels at different temperature and oil/water volume ratios, oil phase (MtBE) stained with Nile red (a) oil : water = 1 : 1, and (b) oil : water = 1.5 : 1. (c) Dynamic contact angles of a water drop on the film of hybrid microgels at different temperatures. (d) The phase inversion diagram of hybrid microgel-stabilized Pickering emulsions with different water fractions at different temperatures. (e) Schematic illustration of the influence of water fraction (*φ*) and temperature on the phase inversion of the as-prepared Pickering emulsions.

Using hydrophobic silica NPs alone can only create w/o Pickering emulsions (Fig. S3†), regardless of the volume ratio of oil/water and correspondingly, the PNIPAM microgel cannot form w/o Pickering emulsions (Fig. S4†). However, the type of Pickering emulsions stabilized by the hybrid microgels could be switched faciley by adjusting the oil/water volume ratios. For example, the as-prepared Pickering emulsion was a w/o type (Fig. 3a and S5†) when the water/oil volume ratio was 1 : 1 at 28 °C, but when the water/oil ratio was 1.5 : 1, it became a o/w type (Fig. 3b). More significantly, altering the temperature *in situ* can also control the state of the resulting Pickering emulsions due to the temperature sensitivity of PNIPAM. As illustrated, when the water/oil ratio was 1 : 1, and the temperature was decreased from 28 °C to 18 °C, the type of the hybrid microgel stabilized was reversible from w/o to o/w (Fig. 3a). Even though the water fraction was higher than that of oil (water : oil = 1.5 : 1), an o/w Pickering emulsion can be successfully converted to a w/o type by increasing the temperature (Fig. 3b), and a higher temperature can further cause the emulsion system to become demulsified (Fig. S6†). The results of the dynamic contact angles in Fig. 3c proved that the hybrid microgel displayed switchable wettability between hydrophobicity and hydrophilicity, allowing for the *in situ* regulated formation of w/o or o/w Pickering emulsions *via* temperature change. Since the total amount of hydrophobic silica NPs on the microgel surface remained constant, it is believed that as the ambient temperature increases, the volume shrinkage of the hybrid microgel leads to denser surface coverage of the hydrophobic sites and thus the hybrid microgel would become more hydrophobic (Fig. 3c). Therefore, the volume fraction of water (*φ*) at the point of phase inversion of the Pickering emulsions stabilized by hybrid microgels can be regulated by temperature change. As summarized in Fig. 3d, Pickering emulsions with varying oil/water volume ratios were prepared for investigating the temperature-controlled type reversion of emulsions. When the temperature increased, the *φ* of emulsion reversion from the o/w to w/o type was correspondingly increased. In comparison to adjusting the oil/water volume ratio, tuning the temperature *in situ* to switch the type of the Pickering emulsion is obviously easier and more accessible. Overall, both the water/oil volume ratio and temperature can be utilized for control over the state of the hybrid microgel-stabilized Pickering emulsions, allowing for the fabrication and adjustment of emulsion systems that meet production needs in a variety of situations, as depicted in Fig. 3e.

**Fig. 4:**
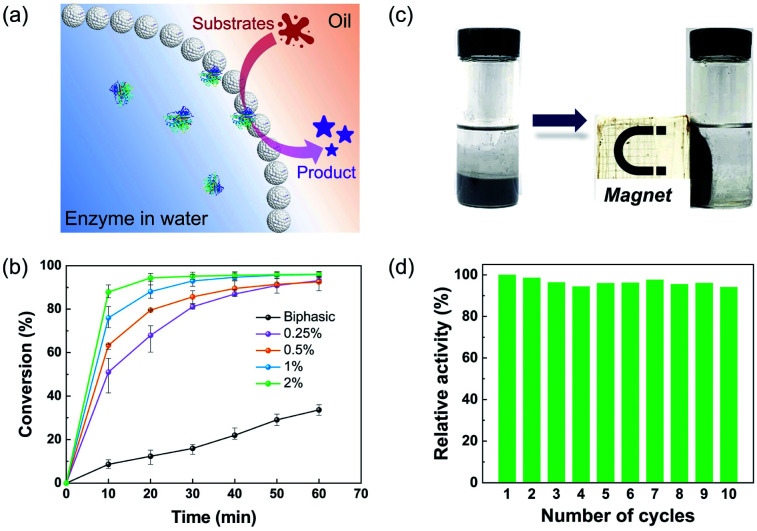
(a) Schematic representation of Pickering emulsions with enzymes in water for interfacial biocatalysis. (b) Plot of the conversion of 1-hexanol and hexanoic acid to ester *versus* the reaction time for free lipase in the biphasic system (●) and lipase encapsulated in Pickering emulsions stabilized by hybrid microgels of different concentrations: 0.25% (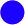), 0.5% (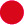), 1% (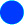), and 2% (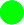). (c) Effect of a static magnetic field on the enzyme-containing w/o Pickering emulsion. (d) Reusability of enzymatic catalysis in the magnetically responsive Pickering emulsion.

Notably, the hybrid microgel in the hydrophobic state had sufficient hydrophobicity to stabilize the w/o Pickering emulsions with a variety of non-polar oils, such as toluene, cyclohexane, and isooctane (Fig. S7†). It highlights the great potential for encapsulation of enzymes within the interior aqueous phase, paving the way for applications in interfacial biocatalysis.^39–41^ The catalytic performance of lipase-loaded Pickering emulsions was initially examined by the esterification of 1-hexanol and hexanoic acid with toluene as the oil phase. In detail, lipase was dissolved and encapsulated in the internal aqueous droplet, while the substrates and product were dissolved in the oil phase, with the catalytic reaction taking place at the w/o interface, as illustrated in Fig. 4a. As a control, a biphasic system containing free lipase was employed. The conversion of the esterification reaction reached more than 90% in four cases employing encapsulated lipases within one hour, but less than 34% for the biphasic group (Fig. 4b). Furthermore, the catalytic performance in Pickering emulsions improved with the increase of the concentration of hybrid microgels, while the enzyme concentration remained constant. Fig. S8† also shows that when the concentration of hybrid microgels increased, the average diameter of the resultant emulsion droplets decreased. As a result, we reasoned that the higher conversion in the Pickering emulsions was due to the greatly enlarged water–oil interfacial area stabilized by hybrid microgels, which significantly improved the mass transfer of substrates and the accessibility of enzymes. Given the high cost of biocatalysts, reusability is an important attribute, and magnetic separation is regarded as a convenient and effective method of recycling catalysts. It is worth noting that magnetic NPs can be easily incorporated into the hybrid microgels during the polymerization process, giving this particulate stabilizer with magnetic responsiveness. As shown in Fig. 4c and d, the aqueous droplets carrying enzymes dispersed in oil can be promptly isolated using a magnet, and then the oil phase containing product can be readily removed and supplied to initiate the next cycle. Even after 10 cycles of consecutive use, the magnetic-responsive Pickering emulsion exhibited exceptional stability, and the catalytic activity of enzymes had no discernible loss.

**Fig. 5:**
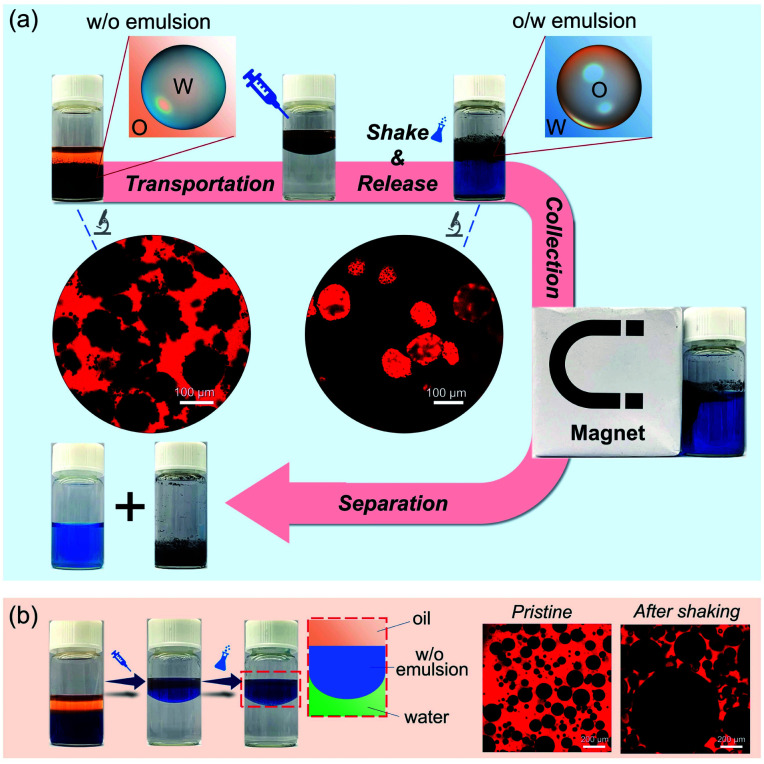
(a) Schematic representation of active release induced by phase inversion. (b) The w/o Pickering emulsion stabilized by hydrophobic silica NPs as the control group for active release. The red color in the CLSM images indicates the Nile red-stained oil phase.

Controlled release is a perpetual hot topic in chemistry and biomedical applications.^42–44^ Herein, attributed to the reversible feature, the as-prepared w/o Pickering emulsion can be employed to protect water-soluble compounds, and then be released by phase inversion. As illustrated in Fig. 5a, methylene blue, as a model substance, was primarily encapsulated in a w/o Pickering emulsion stabilized by hybrid microgels. When the w/o Pickering emulsion was transferred to water, it first floated on top of the water layer that was colorless and transparent. After hand shaking, the w/o Pickering emulsion was instantly converted to an o/w Pickering emulsion, resulting in the rapid release of methylene blue. As a result, the bulk water became blue immediately. Furthermore, the oil droplets can be magnetically collected to realize the separation from bulk water. As shown in Fig. 5b, the w/o Pickering emulsion stabilized by hydrophobic silica NPs was used as a control group, and the w/o emulsion was also suspended on the water, but phase inversion did not occur after shaking and methylene blue could not be released into the water layer.

## Conclusions

In conclusion, we developed a simple approach for hydrophobizing PNIPAM-based microgels and employed them to construct reversible Pickering emulsions. The engineered microgel was composed of a dense PNIPAM shell and a rough surface incorporated with hydrophobic silica NPs, which allowed for swelling-induced changes in the surface wettability. Thus, the phase inversion ratio of oil to water in such microgel-stabilized Pickering emulsions can be simply controlled by temperature. We demonstrated that the hydrophobized microgels were capable of stabilizing w/o emulsions and encapsulating enzymes for interfacial bio-catalysis, and cascade catalysis^45,46^ can be expected by encapsulation of another enzyme in the cavity of the hollow microgel. More importantly, the reversible feature can be exploited to protect hydrophilic actives in the w/o emulsion and to trigger release *via* emulsion phase inversion. Surprisingly, magnetic NPs were effectively integrated as functional sites in the engineered microgel for rapid collection, and further capabilities are envisioned in the future.

## Data availability

Experimental data associated with this article have beenprovided in the ESI.†

## Author contributions

H. Jiang: conceptualization, investigation, methodology, supervision, and writing – review & editing; S. Zhang: data curation, investigation, resources, and writing – original draft; G. Sun, X. Guan, and C. Yang: writing – review & editing; Y. Li: conceptualization, project administration, writing – review & editing, and supervision; T. Ngai: writing – review & editing, supervision, and funding acquisition.

## Conflicts of interest

There are no conflicts to declare.

## Supplementary Material

SC-013-D1SC05398A-s001
