# Supplementary material for: Engineering hybrid microgels as particulate emulsifiers for reversible Pickering emulsions
Source: Chem Sci. 2021 Oct 29;13(1):39–43. doi: 10.1039/d1sc05398a (PMC8694365; doi:10.1039/d1sc05398a)
Supplement: SC-013-D1SC05398A-s001 [file SC-013-D1SC05398A-s001.pdf]

Electronic Supplementary Material (ESI) for Chemical Science.
This journal is © The Royal Society of Chemistry 2021

Supporting Information:

Engineering Hybrid Microgel as Particulate Emulsifier for Reversible Pickering Emulsions

Hang Jiang,‡ [a] Shengwei Zhang,‡ [a] Guanqing Sun,[a] Yunxing Li,*[a] Xin Guan,[b] Cheng Yang,[a] and To Ngai*[a],[b]

[a] The Key Laboratory of Synthetic and Biological Colloids, Ministry of Education, School of Chemical and Material Engineering, Jiangnan University, Wuxi 214122, P. R. China

[b] Department of Chemistry, The Chinese University of Hong Kong, Shatin, N.T., Hong Kong, P. R. China

‡ These authors contributed equally.

E-mail: yunxingli@jiangnan.edu.cn

E-mail: tongai@cuhk.edu.hk

Experimental Procedures

1. Materials

Lipase from *Candida sp.* Expressed in *Aspergillus niger* and Nile red were purchased from Sigma-Aldrich. Hexanoic acid (>99%), 1-hexanol (>99%) were purchased from Tokyo Chemical Industry (TCI). N-Isopropyl acrylamide (NIPAM) (98%), N,N'-methylenebisacrylamide (BIS) (99%), 2,2′-Azobis(2-methylpropionamidine) dihydrochloride (V-50) (97%) were purchased from Aladdin. PolyFluor 570 was purchased from Polysciences. R974 fumed silica and Diethyl Carbonate (DEC) were from Evonik. Absolute ethanol was supplied by Titan Scientific (China) Co., Ltd. Toluene (>99.5%), *Tert*-butyl methyl ether (99%), Sodium dihydngen phoshate (>99%) and sodium phosphate dibasic (>99%) were from Sinopharm Chemical Reagent (China) Co., Ltd. Deionized water was used in all of experiments.

2. Instrumentation

Hybrid microgels and Emulsion droplets were observed with a VHX-1000C 3D microscope (Keyence, China). The morphology of hybrid PNIPAM microgels was observed by scanning electron microscope (S-4800, Hitachi Ltd., Japan) at a voltage of 2kV. All samples were coated with gold before examination. The structures of hybrid microgels and emulsion interface were observed by confocal laser scanning microscope (CLSM) (TCS SP8, Leica, Germany). Emulsion type were identified with upright fluorescence microscope (Nikon 80i, Japan). Contact angle and dynamic interfacial tension measurements were conducted by video optical contact angle measuring instrument (OCA15EC, Dataphysics, Germany). The concentrations of the substates and product were determined using gas chromatograph (Nexis GC-2030, Shimadzu, Japan). Ultrasound experiments was carried with ultrasonic cell crusher (JY92-IIN, Ningbo Licheng Instrument Co., Ltd., China). UV polymerization was carried by the ultraviolet cross linker (Scientz03-II, Ningbo Scientz Biotechnology Co., Ltd., China).

3. Synthesis of hybrid microgels

Fumed silica (0.162 g) was dispersed in 16.2 mL Diethyl Carbonate, N-Isopropyl acrylamide (0.84 g), N,N'-methylenebisacrylamide (0.0252 g) and PolyFluor 570 (0.0002 g) were dissolved in 2.7 mL deionized water, the oil phase and water phase were deoxgenated by bubbling nitrogen gas for 2 min. Then, 2,2′-Azobis(2-methylpropionamidine) dihydrochloride (0.03 g) was add to the water phase. The water phase and oil phase were mixed and emulsified by ultrasonic subsequently. Polymerization was carried out under UV radiation for 6 h. After polymerization, destabilzed the emulsion template and washed the microgels by ethanol for two times, transferred the microgels into water and washed it for at least three times by centrifugation at 8000 rpm. Finally, the microgels were stored in the form of aqueous dispersion. Also, the microgels can also be washed by organic solvents, and stored in the form of organic solvent. Magnetic hybrid microgels were prepared by adding water-soluble Fe_3O_4 (0.189 g) in the water phase before bubbling nitrogen. The distribution of the emulsion template and hybrid microgels measured and calculated by Nano Measurer with 200 emulsion droplets or hybrid microgels in optical micrographs.

4. Preparation of switchable microgel-stabilized emulsions

Aqueous dispersion of the hybrid microgel (2%, w/v) was mixed with *Tert*-butyl methyl ether, following handshaking for 30 s. For exploring the switchable characteristics, varied oil/water volume ratio and temperature were chosen for emulsification. The type of emulsions were determined by "drop test" or fluorescent microscopy.

5. Contact angle and dynamic interfacial tension measurements

Contact angle and dynamic interfacial tension measurements were conducted by video optical contact angle measuring instrument. First, hybrid microgel dispersion (2%, w/v) was coated onto the glass slide to form the film of hybrid microgels. Then, 2 μL of water drop at different temperature was deposited onto the hybrid microgel film by a 0.5 mm syringe needle, and the contact angle was recorded with time. The dynamic interfacial tension of the water-MtBE interface with or without the hybrid microgels was measured via the pendant drop method, with the aqueous droplets suspending in MtBE.

6. Assessment of the performance of interfacial catalysis

The catalytic performance of the biphasic and Pickering systems were evaluated by the esterification of hexanoic acid with 1- hexanol. The w/o Pickering emulsions were produced with enzyme solution (100 μL/mL, 2.5 mL) and toluene dispersion of hybrid microgel (10 mL) with different concentrations (0.25%, 0.5%, 1%, 2%; w/v) by handshaking. Hexanoic acid and 1-hexanol were added into the oil phase to make the concentrations of the substrates reach 0.2 M. Except for the concentrations of hybrid microgel, the formulation of all groups was same. The esterification reactions were performed at room temperature, and 10 μL aliquots were extracted every ten minutes, centrifuged and analyzed by gas chromatography (GC). The cycles of enzymatic catalysis were investigated by reuse of the Pickering emulsion stabilized with magntic hybrid microgel toluene dispersion (2% w/v, 40 min per cycle). After each reaction cycle, the w/o droplets containing enzyme can be separated by magnet fast and easily,and the oil phase containing substrate and product can be removed conveniently. Then, the residual reactants were removed by rinsing with fresh toluene, and a new substates solution was added for the next cycles. The temperature program for GC setup was as follow: start temperature 80 °C, hold for 0.5 min, temperature increase at 10 °C/min from 80 to 200 °C, hold for 2 min.

Results and Discussion

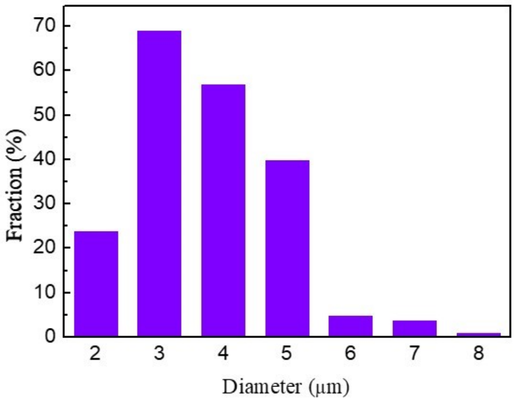

Figure S1. Size distribution of w/o Pickering emulsion template.

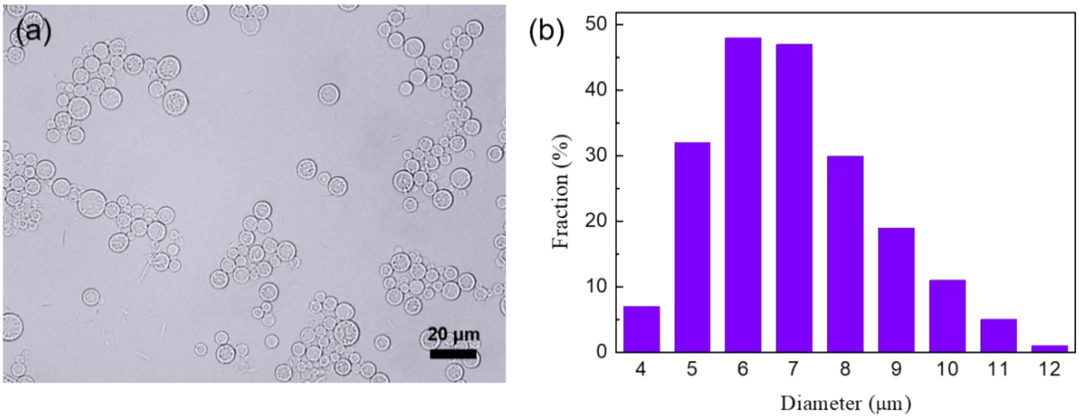

Figure S2. Optical microscope image (a) and size distribution (b) of hybrid microgels dispersed in water.

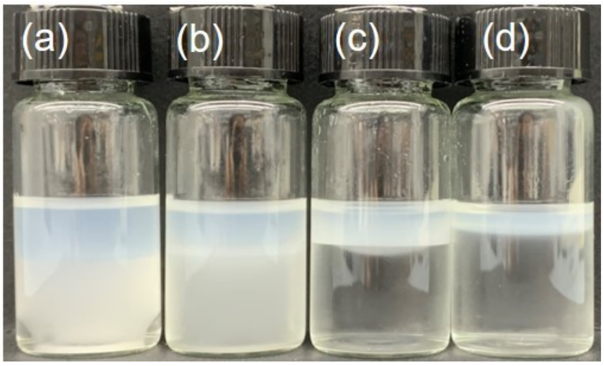

Figure S3. Picture of hydrophobic silica NPs stabilized Pickering emulsions with varied water/oil ratios (v/v), (a) 1: 1, (b) 2: 1, (c) 3: 1, (d) 5: 1.

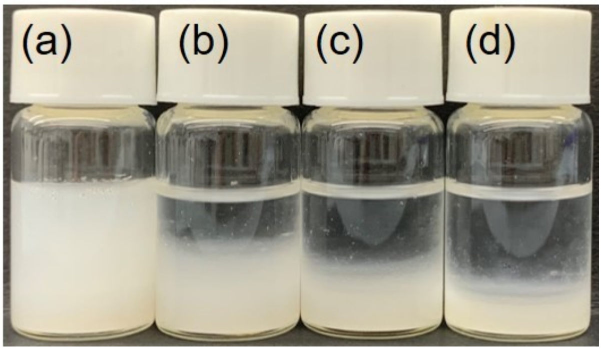

Figure S4. Picture of PNIPAM microgel stabilized Pickering emulsions with varied water/oil ratios (v/v), (a) 1: 1, (b) 1: 1.5, (c) 1: 2, (d) 1: 3.

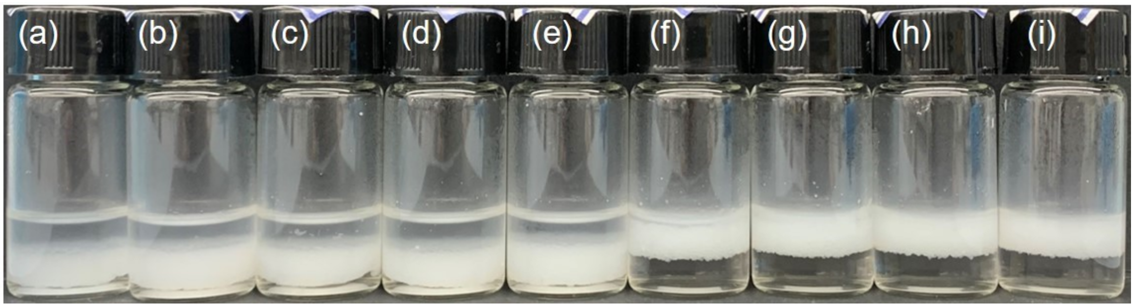

Figure S5. Picture of hybrid microgel stabilized Pickering emulsions with varied water/oil ratios (v/v), (a) 1: 1, (b) 1.04: 0.96, (c) 1.08: 0.92, (d) 1.12: 0.88, (e) 1.16: 0.84, (f) 1.2: 0.8 (1.5: 1, phase inverse point), (g) 1.24: 0.76, (h) 1.28: 0.72, (i) 1.32: 0.68.

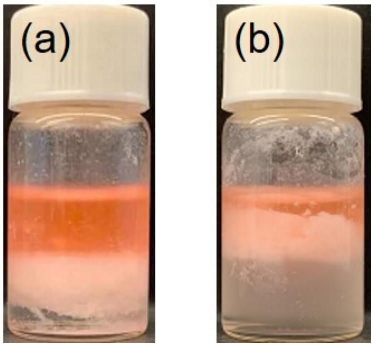

Figure S6. Pictures of demulsified emulsions stabilized by hybrid microgel with different water/oil ratio (v/v), (a) 1: 1, 45 °C, (b) 1.5:1, 42 °C.

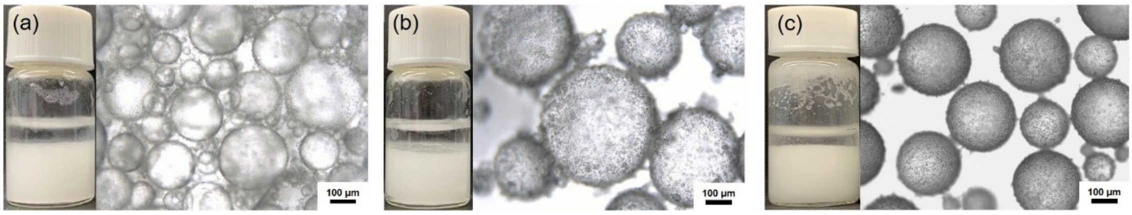

Figure S7. Pictures and optical microscopy images of hybrid microgel stabilized Pickering emulsions with different oils, (a) isooctane, (b) cyclohexane, (c) toluene.

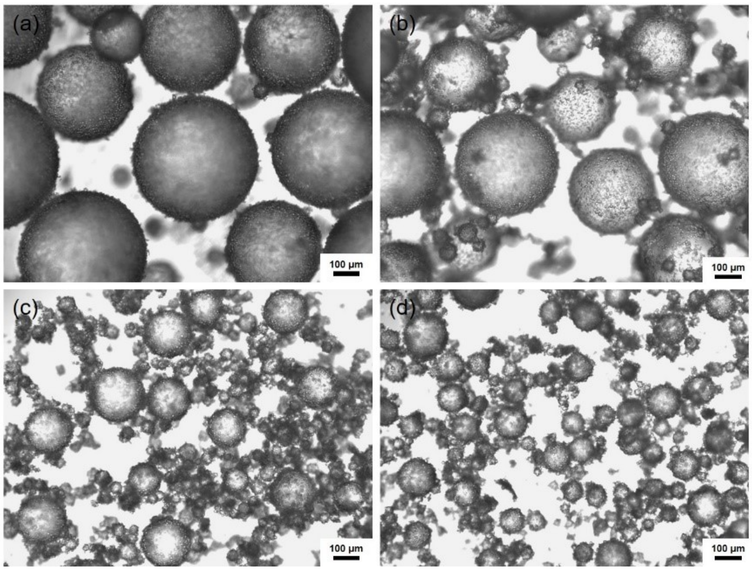

Figure S8. Optical microscopy images of hybrid microgel stabilized Pickering emulsions with varied concentrations, (a) 0.25%, (b) 0.5%, (c) 1%, (d) 2%.
